# The role of IP_3_ receptors and SERCA pumps in restoring working memory under amyloid β induced Alzheimer's disease: a modeling study

**DOI:** 10.3389/fncom.2025.1643547

**Published:** 2025-07-22

**Authors:** Ziyi Huang, Lei Wang

**Affiliations:** Scholastic Excellence Research Center, Wuxi Dipont School of Arts and Science, Wuxi, China

**Keywords:** working memory, Alzheimer's disease, spiking network, IP_3_ receptors, SERCA pumps

## Abstract

Memory impairment is a prevalent symptom in patients with Alzheimer's disease (AD), with working memory loss being the most prominent deficit. Recent experimental evidence suggests that abnormal calcium levels in the Endoplasmic Reticulum (ER) may disrupt synaptic transmission, leading to memory loss in AD patients. However, the specific mechanisms by which intracellular calcium homeostasis influences memory formation, storage, and recall in the context of AD remain unclear. In this study, we investigate the effects of intracellular calcium homeostasis on AD-related working memory (WM) using a spiking network model. We quantify memory storage by measuring the similarity between images during the training and testing phases. The model results indicate that ~90% of memory can be stored in the WM network under normal conditions. In contrast, the presence of amyloid beta (*A*β), associated with AD, significantly reduces this similarity, allowing only 54%-58% of memory to be stored, this alteration trend is consistent with previous experimental findings. Further analysis reveals that downregulating the activation of inositol triphosphate (*IP*_3_) receptors and upregulating the activation of the sarco-endoplasmic reticulum *Ca*^2+^ ATPase (*SERCA*) pumps can enhance memory performance, achieving about 78% and 77%, respectively. Moreover, simultaneously manipulating both *IP*_3_ and *SERCA* activations can increase memory capacity to around 81%. These findings suggest several potential therapeutic targets for addressing memory impairment in *A*β aggregation induced AD patients. Additionally, our network model could serve as a foundation for exploring further mechanisms that modulate memory dysfunction at the genetic, cellular, and network levels.

## 1 Introductions

Alzheimer's disease (AD) is the most prevalent neurodegenerative disorder and the leading cause of dementia worldwide. It is affecting more than 40 million people globally, as this number is constantly increasing (Kim et al., 2024). AD is commonly associated with the accumulation of amyloid-beta (*A*β) plaques and tau tangles. Cognitive symptoms such as working memory (WM) loss are often detected before these pathological hallmarks (Breijyeh and Karaman, 2020). The early dysfunction of working memory (i.e., the ability to temporarily store and process information) shows that defects in neuronal pathways may result in the initial cognitive deficits before the major neurodegenerations occur.

Recent advances in neuroscience research demonstrate that astrocyte, the previously considered supporting cell (Kimelberg and Nedergaard, 2010; Farhy-Tselnicker and Allen, 2018), plays an important role in WM via synapse modulations (Gordleeva et al., 2021). Astrocytes respond to neuronal activities by producing intracellular inositol triphosphate (*IP*_3_), causing calcium (*Ca*^2+^) release from internal stores. The *Ca*^2+^ elevation triggers the increase of gliotransmitters release, which enhances the synaptic connections, forming the basis of short-term memory formation. This feedback loop has been tested to successfully model the encoding and retrieval of WM under a biologically plausible network (Gordleeva et al., 2021).

However, this intricate mechanism became vulnerable in the situation of AD. *A*β oligomers is studied to interfere with intracellular *Ca*^2+^ homeostasis by enhancing membrane leak, over-activating *IP*_3_ receptors and suppressing the activity of sarco-endoplasmic reticulum *Ca*^2+^ ATPase (*SERCA*) pumps, leading to persistent intracellular *Ca*^2+^ elevation and signaling irregularities (Latulippe et al., 2018). This abnormality leads to the unstable intracellular environment while also impairs the astrocytic capacity on modulating working memory, which might be the reason of WM damage.

Most research on the causes and treatments of AD has been conducted through experimental methods, which are often rigorous and time-consuming. Given this challenge, computational approaches have emerged as a viable alternative (Moravveji et al., 2024). In recent decades, numerous network models have been developed to explore potential mechanisms related to the causes and treatments of AD, addressing areas such as disease progression (Chamberland et al., 2024; Bertsch et al., 2017), pathogenesis (Puri and Li, 2010), the effects of specific proteins (Helal et al., 2019, 2014), and mitochondrial dysfunction (Toglia et al., 2018). Many of these studies have utilized non-spiking neuron models; however, spiking signals are intrinsic to neurons and can be reliably transmitted over long distances in brain regions affected by AD. Therefore, this study employs spiking neuron models as the primary functional units in constructing the network.

In this study, we construct a computational network model to examine how intracellular calcium homeostasis affects the formation, impairment, and restoration of WM under *A*β-induced AD conditions. The network comprises two cell types: spiking excitatory neurons and non-spiking astrocytes. The neurons are primarily responsible for generating population spiking activity, while the astrocytes are mainly involved in producing various calcium signals. Model results indicate that the presence of *A*β impairs WM performance by significantly increasing *Ca*^2+^ concentrations. Conversely, downregulating *IP*_3_ activation and upregulating *SERCA* activation, either separately or simultaneously, can help restore WM performance to some extent.

## 2 Model descriptions

In this study, we introduce a biologically plausible spiking neuron-astrocyte network that simulates WM through local synaptic modulations. Neurons generate spikes in response to external stimuli, releasing glutamates into the extracellular space. Surrounding astrocytes detect these glutamates, activating internal *IP*_3_ and *Ca*^2+^ signaling cascades. When astrocytic *Ca*^2+^ exceeds a critical threshold, gliotransmitters are released, transiently enhancing synaptic weights in the stimulated neuronal subnetwork. This temporary potentiation supports cue-based memory retrieval during test phases. In the following subsections, we detail the model components responsible for simulating this loop, including the neuron dynamics, astrocyte calcium signaling, *A*β modulation, and memory performance metrics.

Architecture and cell units of our network model are inspired and adapted from Gordleeva et al. (2021). Based on the spiking network, we employed three additional elements: *A*β-dependent calcium flows, calcium-dependent variations of synaptic weight combined with *A*β modulations, and negative components in describing the activation of *IP*_3_. The first two elements are used to introduce the influence of *A*β. The larger the *A*β value, the more severe the AD and the worse the WM performance. The last element is used to balance the variation of *IP*_3_.

### 2.1 Neuron model

Spiking dynamics of single neuron is described using the Izhikevich model (Izhikevich, 2003). Due to its simplicity and computational efficient, this neuron model has been widely used to study population activities of neurons, e.g., synchronization and oscillation (Khoshkhou and Montakhab, 2018).

Mathematical expressions of the Izhikevich model are:


(1) $$\begin{array}{l} \frac{dV\left(i,j\right)}{dt}=0.04{\left(V\left(i,j\right)\right)}^{2}+5V\left(i,j\right)-U\left(i,j\right)+140+I_{app}\left(i,j\right)\\ \;+I_{syn}\left(i,j\right)\\ \frac{dU\left(i,j\right)}{dt}=a\left(bV\left(i,j\right)-U\left(i,j\right)\right) \end{array}$$

with the auxiliary after-spike resetting:


(2) $$\begin{array}{lll} \text{if }V\left(i,j\right) & \geq & 30\text{ mV},\text{ then }\{\begin{array}{l} V\left(i,j\right)\;\leftarrow c\\ U\left(i,j\right)\;\leftarrow U\left(i,j\right)+d \end{array} \end{array}$$

Here, *V* represents the transmembrane potential, while *U* denotes a membrane recovery variable that provides negative feedback to *V*. The indices (*i, j*) indicate the corresponding neuron. *c* is the resting potential, and *a*, *b*, *d* are dimensionless parameters. *I*_*app*_ refers to the applied currents to the respective neurons, which will be explained further below.

Synaptic currents that neurons receive is expressed as Gordleeva et al. (2021):


(3) $$\begin{array}{l} I_{syn}\left(i,j\right)=\overset{N\left(i,j\right)}{\underset{k=1}{\sum }}\frac{g_{syn}\left(i,j\right)\left(E_{syn}-V\left(i,j\right)\right)}{1+\exp\left(-\frac{V_{pre}^{k}}{k_{syn}}\right)} \end{array}$$

Here, *N* is the total number of presynaptic neurons, *E*_*syn*_ is the reversal potential for the synapse, *V*_*pre*_ denotes the membrane potential of the presynaptic neuron, and *k*_*syn*_ is slope of the synaptic activation function. The parameter *g*_*syn*_ describes the synaptic strength (Gordleeva et al., 2021):


(4) $$\begin{array}{l} g_{syn}\left(i,j\right)=\;\eta +v_{ca}\left(m,n\right) \end{array}$$

Here (*m, n*) denotes the index of the corresponding astrocyte that modulates the synaptic currents of neuron (*i, j*). η represents the synaptic weight without astrocyte influence, while *v*_*ca*_ denotes the astrocyte-induced modulation of synaptic strength.

Specific values of these parameters are given in Table 1.

**Table 1:** Parameter values in the neuron model.

**Parameter**	**Description**	**Value**
***a***	Time scale of the recovery variable	0.1
***b***	Sensitivity of the recovery variable to the subthreshold fluctuation of membrane potential	0.2
***c***	After-spike reset value of the membrane potential	−65 mV
***d***	After-spike reset of the recovery variable	2
***N***	Number of input connections per each neuron	40
***η***	Synaptic weight without astrocyte inputs	0.025
***E*_*syn*_**	Synaptic reversal potential for excitatory synapse	0 mV
***k*_*syn*_**	Slope of the synaptic activation function	0.2 mV

### 2.2 Astrocyte model

**Figure 1:**
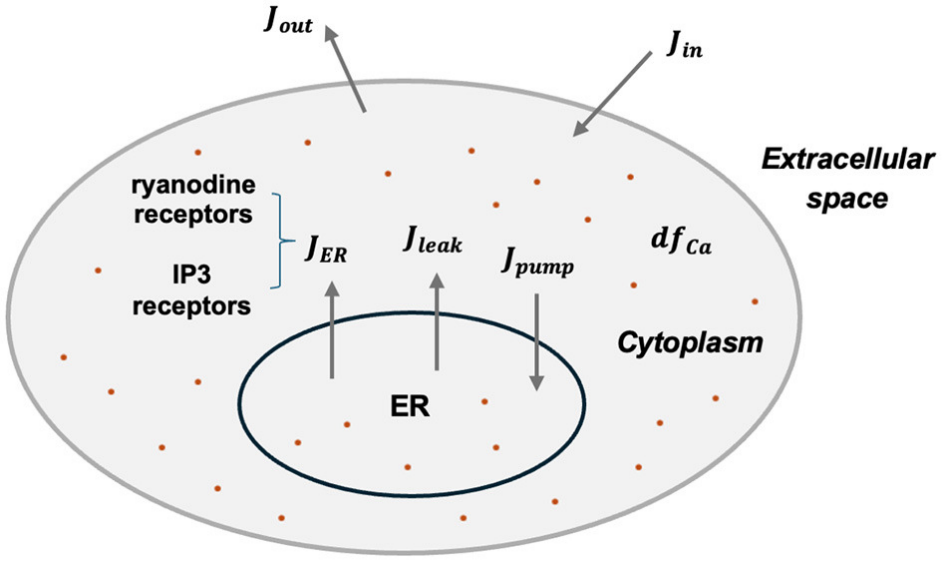
Schematic diagram of intracellular calcium exchanges.

As mentioned above, astrocytes in our network primarily generate various calcium signals. Following the method used in the previous study (Gordleeva et al., 2021), a mean-field approach is employed to describe the emergence of *Ca*^2+^ signals, as shown in Equation 5 and Figure 1.


(5) $$\begin{array}{l} \frac{d\left[Ca^{2+}\right]\left(m,n\right)}{dt}=J_{ER}\left(m,n\right)-J_{pump}\left(m,n\right)+J_{leak}\left(m,n\right)\\ \;+J_{in}\left(m,n\right)-J_{out}\left(m,n\right)+df_{Ca}\left(m,n\right)\\ \;\frac{dh\left(m,n\right)}{dt}=a_{2}(d_{2}\frac{IP_{3}\left(m,n\right)+d_{1}}{IP_{3}\left(m,n\right)+d_{3}}\left(1-h\left(m,n\right)\right)\\ \;-\left[Ca^{2+}\right]\left(m,n\right)\;h\left(m,n\right)) \end{array}$$

Here *Ca*^2+^ indicate the intracellular calcium concentration, *h* is the fraction of the activated *IP*_3_ receptors on the ER surface. Detailed expressions of each flux are:


(6) $$\begin{array}{l} \;J_{ER}=c_{1}v_{1}{\left[Ca^{2+}\right]}^{3}h^{3}IP_{3}^{3}\frac{\left(c_{0}/c_{1}-\left(1+1/c_{1}\right)\left[Ca^{2+}\right]\right)}{{\left(\left(IP_{3}+d_{1}\right)\left(\left[Ca^{2+}\right]+d_{5}\right)\right)}^{3}}\\ J_{pump}=\;\gamma \frac{v_{3}{\left[Ca^{2+}\right]}^{2}}{k_{3}^{2}+{\left[Ca^{2+}\right]}^{2}}\\ \;J_{leak}=c_{1}v_{2}\left(c_{0}/c_{1}-\left(1+1/c_{1}\right)\left[Ca^{2+}\right]\right)\\ \;J_{in}=\frac{v_{6}IP_{3}^{2}}{k_{2}^{2}+IP_{3}^{2}}+v_{x}\\ \;J_{out}=k_{1}\left[Ca^{2+}\right] \end{array}$$

Here *J*_*ER*_ represents the *Ca*^2+^ flux from the ER to the cytoplasm through *IP*_3_ receptors and ryanodine receptors (*RyR*). *J*_*pump*_ denotes the *Ca*^2+^ flux pumped back into the ER via the *SERCA*, while *J*_*leak*_ indicates the leakage flux from the ER to the cytoplasm. *J*_*in*_ and *J*_*out*_ describe the calcium exchange with extracellular space.

Activation dynamics of *IP*_3_ is expressed as Gordleeva et al. (2021) and Wagner et al. (2004):


(7) $$\begin{array}{l} \frac{dIP_{3}\left(m,n\right)}{dt}=\frac{IP_{3}^{*}-IP_{3}\left(m,n\right)}{\tau_{IP_{3}}}+\lambda (J_{PLC\delta }\left(m,n\right)+J_{glu}\left(m,n\right)\\ \;+df_{IP_{3}}\left(m,n\right))-kv_{4}\frac{J_{Kinase}\left(m,n\right)-J_{Phosphatase}\left(m,n\right)}{\lambda } \end{array}$$

Here $IP_{3}^{*}$ denotes the steady-state concentration of the *IP*_3_ receptors, while τ_*I*_*P*__3__ is the rate constant for *IP*_3_ loss. λ is a control parameter used to regulate various elements in *IP*_3_ dynamics. *J*_*PLCδ*_ describes the production of *IP*_3_ by phospholipase *C*δ (*PLC*δ), expressed as Gordleeva et al. (2021):


(8) $$\begin{array}{l} J_{PLC\delta }=\frac{v_{4}\left(\left[Ca^{2+}\right]+\left(1-\alpha \right)k_{4}\right)}{\left[Ca^{2+}\right]+k_{4}} \end{array}$$

*J*_*glu*_ describes the production of *IP*_3_ induced by glutamate in response to neuronal activities, which is modeled as Gordleeva et al. (2021):


(9) $$\begin{array}{l} J_{glu}=\;\{\begin{array}{c} A_{glu},\text{ if }t_{0}<t\leq t_{0}+t_{glu}\\ 0,\text{ otherwise } \end{array} \end{array}$$

Here *A*_*glu*_ represents the amplitude of glutamate contributing to the production of *IP*_3_, while *t*_*glu*_ denotes the periods when the total level of glutamate from all synapses reaches a given threshold:


(10) $$\begin{array}{l} \left(\frac{1}{N_{a}}\underset{\left(i,j\right)\in N_{a}}{\sum }\left[G\left(i,j\right)>G_{thr}\right]\right)>F_{act} \end{array}$$

Here *N*_*a*_ represents the number of neurons connected to a single astrocyte, *G*_*thr*_ = 0.7 is the threshold, and [*x*] denotes the Iverson bracket. *F*_*act*_ = 0.5 indicates the fraction of synchronously spiking neurons within the total neuronal ensemble associated with the astrocyte. *G* refers to the amount of glutamate, which will be explained further below.

*J*_*Kinase*_ and *J*_*Phosphatase*_ are currents for *IP*_3_ degradation, which are expressed as Wagner et al. (2004):


(11) $$\begin{array}{l} J_{Kinase}=\left(1-\rho \right)kv_{1}\frac{IP_{3}}{IP_{3}+2.5}+\rho \cdot kv_{2}\frac{IP_{3}}{IP_{3}+0.5} \end{array}$$

(12) $$\begin{array}{l} J_{Phosphatase}=kv_{3}\frac{IP_{3}}{IP_{3}+30} \end{array}$$

(13) $$\begin{array}{l} \rho =\frac{\left[Ca^{2+}\right]}{\left[Ca^{2+}\right]+0.39} \end{array}$$

The currents *df*_*Ca*_ in Equation 5 and *df*_*I*_*P*__3__ in Equation 7 represent the diffusion of *Ca*^2+^ ions and *IP*_3_ molecules via gap junctions between astrocytes, expressed as follows (Gordleeva et al., 2021):


(14) $$\begin{array}{l} \;df_{Ca}\left(m,n\right)=d_{Ca}\left(\Delta \left[Ca^{2+}\right]\right)\left(m,n\right)\\ df_{IP_{3}}\left(m,n\right)=d_{IP_{3}}\left(\Delta IP_{3}\right)\left(m,n\right) \end{array}$$

Here *d*_*Ca*_ and *d*_*I*_*P*__3__ represent the diffusion rate of the *Ca*^2+^ and *IP*_3_, respectively. Following a previous study (Gordleeva et al., 2021), we assume that each astrocyte is diffusively coupled only with its four nearest neighbors:


(15) $$\begin{array}{l} \left(\Delta \left[Ca^{2+}\right]\right)\left(m,n\right)=\left(\Delta \left[Ca^{2+}\right]\right)\left(m+1,n\right)\\ \;+\left(\Delta \left[Ca^{2+}\right]\right)\left(m-1,n\right)\\ \;+\left(\Delta \left[Ca^{2+}\right]\right)\left(m,n+1\right)\\ \;+\left(\Delta \left[Ca^{2+}\right]\right)\left(m,n-1\right)-4\left(Ca^{2+}\right)\left(m,n\right) \end{array}$$

(16) $$\begin{array}{l} \;\left(\Delta IP_{3}\right)\left(m,n\right)=\left(\Delta IP_{3}\right)\left(m+1,n\right)+\left(\Delta IP_{3}\right)\left(m-1,n\right)\\ \;+\left(\Delta IP_{3}\right)\left(m,n+1\right)+\left(\Delta IP_{3}\right)\left(m,n-1\right)\\ \;-4\left(IP_{3}\right)\left(m,n\right) \end{array}$$

Specific values of these parameters are given in Table 2.

**Table 2:** Parameter values in astrocyte model.

**Parameter**	**Description**	**Value**
***v*_1_**	Max *Ca*^2+^ flux	6 *s*^−1^
***v*_2_**	*Ca*^2+^ leak flux constant	0.11 *s*^−1^
***v*_3_**	Max *Ca*^2+^ uptake	2.2 μ*M*/*s*
***v*_4_**	Max rate of *IP*_3_ production	0.3 μ*M*/*s*
***v*_6_**	Max rate of activation-dependent *Ca*^2+^ influx	0.2 μ*M*/*s*
***v*_*x*_**	*Ca*^2+^ influx scaling factor	0.025 μ*M*/*s*
***c*_0_**	Total *Ca*^2+^ in the cytoplasm	2.0 μ*M*
***c*_1_**	Ratio of ER volume to cytoplasm volume	0.185
***d*_1_**	Dissociation constant for *IP*_3_	0.13 μ*M*
***d*_2_**	Dissociation constant for *Ca*^2+^ inhibition	1.049 μ*M*
***d*_3_**	Receptor dissociation constant for *IP*_3_	943.4 *nM*
***d*_5_**	*Ca*^2+^ activation constant	82 *nM*
***k*_1_**	Rate constant of *Ca*^2+^ extrusion	0.5 *s*^−1^
***k*_2_**	Half-saturation constant for agonist-dependent *Ca*^2+^ entry	1 μ*M*
***k*_3_**	Activation constant for *ATP*−*Ca*^2+^ pump	0.1 μ*M*
***k*_4_**	Dissociation constant for *Ca*^2+^ stimulation of *IP*_3_ production	1.1 μ*M*
***kv*_1_**	Max rate constant at low *Ca*^2+^	0.001 μ*M*/*s*
***kv*_2_**	Max rate constant at high *Ca*^2+^	0.005 μ*M*/*s*
***kv*_3_**	Max rate constant (phosphatase)	0.02 μ*M*/*s*
***kv*_4_**	*IP*_3_ production rate scaling factor	10/0.083
$\text{I}P_{3}^{*}$	Steady state concentration of *IP*_3_	0.16 μ*M*
***d*_*ca*_**	*Ca*^2+^ diffusion rate	0.05 *s*^−1^
***d*_*I*_*P*__3__**	*IP*_3_ diffusion rate	0.1 *s*^−1^
***A*_*glu*_**	Rate of *IP*_3_ production through glutamate	5 μ*M*/*s*

### 2.3 Neuron-astrocyte network

**Figure 2:**
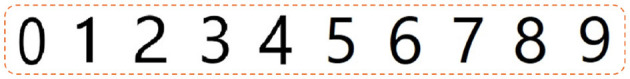
Image stimulus patterns.

As described in Gordleeva et al. (2021), the network model for WM comprises three layers: the input layer, the neurons layer, and the astrocytes layer. The input layer consists of two types of stimuli: (1) image signals labeled {“**0**″, “**1**″, “**2**″, “**3**″, “**4**″, “**5**″, “**6**″, “**7**″, “**8**″, “**9**″} (Figure 2), which are responsible for memory training and testing, and (2) background noise signals, that generate low-rate spontaneous spikes.

The neurons layer contains 79 × 79 neurons, which receive stimuli from the input layer and project to the astrocytes layer. The third layer consists of 26 × 26 astrocytes, with each astrocyte projecting back to neurons in the second layer.

The architecture of synaptic connections among neurons is random; specifically, each neuron connects to *N* = 40 local postsynaptic target neurons via excitatory chemical synapses, with these neurons chosen randomly from the neurons layer. In contrast, the synaptic connections among astrocytes are deterministic, with each astrocyte connecting to its four nearest neighbors via gap junctions (Gordleeva et al., 2021).

Additionally, each astrocyte connects with *N*_*a*_ = 16 (*i*.*e*., 4 × 4) neurons through reciprocal excitatory chemical synapses. Thus, the synaptic strength in Equation 4 has a *Ca*^2+^ dependent component *v*_*Ca*_, which can be expressed as:


(17) $$\begin{array}{l} v_{Ca}=v_{Ca}^{*}\Theta \left({\left[Ca^{2+}\right]}^{\left(m,n\right)}-{\left[Ca^{2+}\right]}_{thr}\right) \end{array}$$

Here $v_{Ca}^{*}$ denotes the strength of astrocyte-induced modulation of synaptic weight, Θ(*x*) is the Heaviside step-function, and ${\left[Ca^{2+}\right]}_{thr}$ is the threshold.

In Equation 10, the amount of glutamate *G* is characterized as Gordleeva et al. (2021):


(18) $$\begin{array}{l} \frac{dG\left(i,j\right)}{dt}=-\alpha_{glu}G\left(i,j\right)+k_{glu}\Theta \left(V\left(i,j\right)-30\right) \end{array}$$

Here α_*glu*_ represents the glutamate clearance constant, while *k*_*glu*_ indicates the efficacy of the release. Similar to Equation 17, Θ(*x*) is the Heaviside step-function.

Specific values of relevant parameters are given in Table 3.

**Table 3:** Parameter values in neuron-astrocyte network.

**Parameter**	**Description**	**Value**
***N*_*a*_**	Number of neurons connecting with one astrocyte	16
**α_*glu*_**	Glutamate clearance constant	10 *s*^−1^
***k*_*glu*_**	Efficacy of glutamate release	600 μ*M*/*s*
${\text{v}}_{ca}^{*}$	Strength of astrocyte–induced modulation of synaptic weight	0.5
${\left[Ca^{2+}\right]}_{thr}$	Threshold concentration of *Ca*^2+^ for the astrocytic modulation of synapse	0.15 μ*M*

### 2.4 Stimulation protocol

Our stimulation protocol follows the delayed match-to-sample task (DMS), which is commonly used in experimental studies of memory formation and recall. During the training phase, an image labeled with a specific digit is presented for 200 *ms* (500 *ms*-700 *ms*), followed by a 700 *ms* break (700 *ms*-1,400 *ms*). After this, two non-match images are displayed, each for 150 *ms* (1,400 *ms*-1,550 *ms* and 1,800 *ms*-1,950 *ms*), with a 250 *ms* break in between (1,550 *ms*-1,800 *ms* and 1,950 *ms*-2,200 *ms*). Finally, the matching stimulus appears for 150 *ms* (2,200 *ms*-2,350 *ms*) to test the network's memory recall capability. The total duration of the simulation experiment is 3,000 *ms*. Detailed stimulus information during training and testing phases can refer to Gordleeva et al. (2021). A demonstration of a sample stimulus current and the corresponding neuronal activities during the training and test phases is presented in Supplementary Figure 17.

### 2.5 Memory performance matrices

To quantify the amount of memory that can be stored, we use a measure calculated based on the similarity between a recalled image and the sample image. The similarity ranges from 0 to 1, with larger values indicating better WM performance.


(19) $$\begin{array}{l} M_{ij}\left(t\right)=I\left[\left(\sum_{k=t-w}^{t}I\left[V_{ij}\left(k\right)>thr\right]\right)>0\right]\\ \;C\left(t\right)=\frac{1}{2}(\frac{1}{\left|P\right|}\sum_{\left(i,j\right)\in P}M_{ij}\left(t\right)+\frac{1}{W\cdot H-\left|P\right|}\sum_{\left(i,j\right)\notin P}(1\\ \;-M_{ij}(t)))\\ \;C_{P}=\frac{1}{\left|T_{P}\right|}\underset{t\in T_{P}}{\max}C(t) \end{array}$$

Here *w* = 1 *ms* represents the time step, *thr* denotes the spiking threshold of the neuron, *P* is the set of pixels belonging to the sample image, and *W* and *H* are the network dimensions. *I* is the indicator function, and *T*_*P*_ is the set of frames within the tracking range of pattern *P*. For more details (see Gordleeva et al., 2021).

### 2.6 Introduction of **Aβ** to calcium flows

The effect of *A*β on calcium dynamics is reflected in its influence on the currents *J*_*in*_ and *J*_*ER*_. Following a previous study (Latulippe et al., 2018), *J*_*in*_ and *J*_*ER*_ with the addition of *A*β are expressed as:


(20) $$\begin{array}{l} J_{in}=\frac{v_{6}IP_{3}^{2}}{k_{2}^{2}+IP_{3}^{2}}+v_{x}+A_{\beta }^{4}\\ J_{ER}=c_{1}v_{1}{\left[Ca^{2+}\right]}^{3}h^{3}IP_{3}^{3}\frac{\left(c_{0}/c_{1}-\left(1+1/c_{1}\right)\left[Ca^{2+}\right]\right)}{{\left(\left(IP_{3}+d_{1}\right)\left(\left[Ca^{2+}\right]+d_{5}+0.02A_{\beta }\right)\right)}^{3}} \end{array}$$

Here *A*_β_ represents a fixed level of *A*β concentration.

### 2.7 Calcium-dependent change of synaptic weights with **Aβ** modulations

Previous experimental results indicate that high concentrations of *Ca*^2+^ can promote the release probability of neurotransmitters, thereby strengthening synaptic connections between neurons (Neher and Sakaba, 2008). However, excessive levels of *Ca*^2+^ can disrupt synaptic connections by inducing irreversible excitotoxic injury (Vermma et al., 2022). In our model, Equation 21 is employed to prevent exaggerated *Ca*^2+^ concentrations.


(21) $$\begin{array}{l} w_{a}=\frac{1.2}{1+\exp\left(\frac{1-12.1\left[Ca^{2+}\right]}{0.2}\right)}\exp\left(-\left[Ca^{2+}\right]\right) \end{array}$$

Another experiment demonstrated that the accumulation of *A*β negatively affects *Ca*^2+^ levels (Toglia et al., 2018). Therefore, Equation 22 is introduced to simulate this variation.


(22) $$\begin{array}{l} w_{b}=\{\begin{array}{c} 1+\exp\left(-2A_{\beta }\right)w_{a}\text{ if }\left[Ca^{2+}\right]\;\leq 0.15\\ \exp\left(-2A_{\beta }\right)w_{a}\text{ else } \end{array} \end{array}$$

Here *A*_β_ represents a fixed level of *A*β concentration. Based on the value of *w*_*b*_, we have $v_{Ca}^{*}=w_{b}\;*\;v_{Ca}^{*}$.

All simulations were performed using the Matlab software (Matlab R2021a), and the fourth-order Runge-Kutta algorithm was employed to calculate the values of different variables with a time integration step of 0.1 *ms*.

## 3 Model results

In this section, we present the functional outcomes of the neuron-astrocyte network model under both normal and AD-like conditions. We begin by illustrating how the intact system encodes, maintains, and successfully retrieves a single stimulus, highlighting the role of astrocyte-mediated synaptic modulation in WM. Next, we analyze memory performance metrics across varying levels of *A*β pathology and under parameter modulations targeting *IP*_3_ production and *SERCA* activity. These results provide valuable insights into the effects of calcium dysregulation on WM and emphasize potential strategies for restoring cognitive function in *A*β-induced AD.

### 3.1 WM network performance under normal conditions

**Figure 3:**
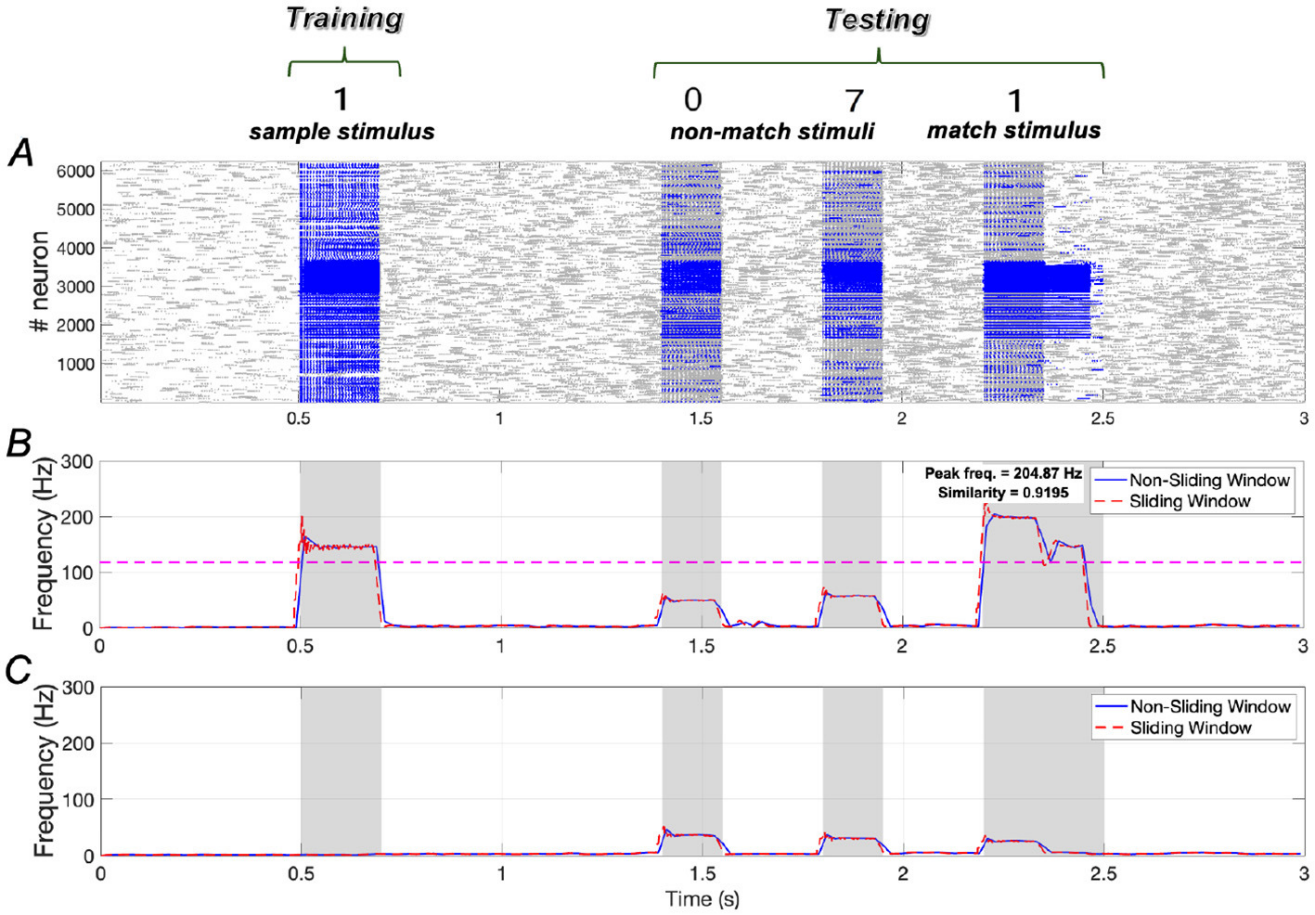
Neuronal responses in WM network under normal conditions. **(A)** Raster plots of spikes for all the neurons during different phases; **(B, C)** Average frequencies of stimulus-specific and unspecific neurons using different time windows, respectively (bin = 20 *ms*).

In the absence of *A*β (indicating no AD symptoms), the WM network performs effectively. Results shown in Figure 3 illustrate that the training stimulus “**1**” can be successfully recalled during the testing period after two non-match stimuli (“**0**” and “**7**”). Quantitative results in terms of similarity and peak frequency indicate that the similarity of neuronal responses to the sample stimulus and the match stimulus reaches ~0.9195. Additionally, the peak frequency of neurons responding to the match stimulus is comparable to that of neurons responding to the sample stimulus.

The dotted pink line in Figure 3B is determined by evaluating each stimulus from the set {“**0**″, “**2**″, “**3**″, “**4**″, “**5**″, “**6**″, “**7**″, “**8**″, “**9**″} as the match stimulus and calculating the maximum peak frequency among these stimuli (see Supplementary Figure 18). If the peak frequency of neurons under the match stimulus “**1**” exceeds this line, we consider neuronal activity under “**1**” to be distinguishable from the activities elicited by the other stimuli.

**Figure 4:**
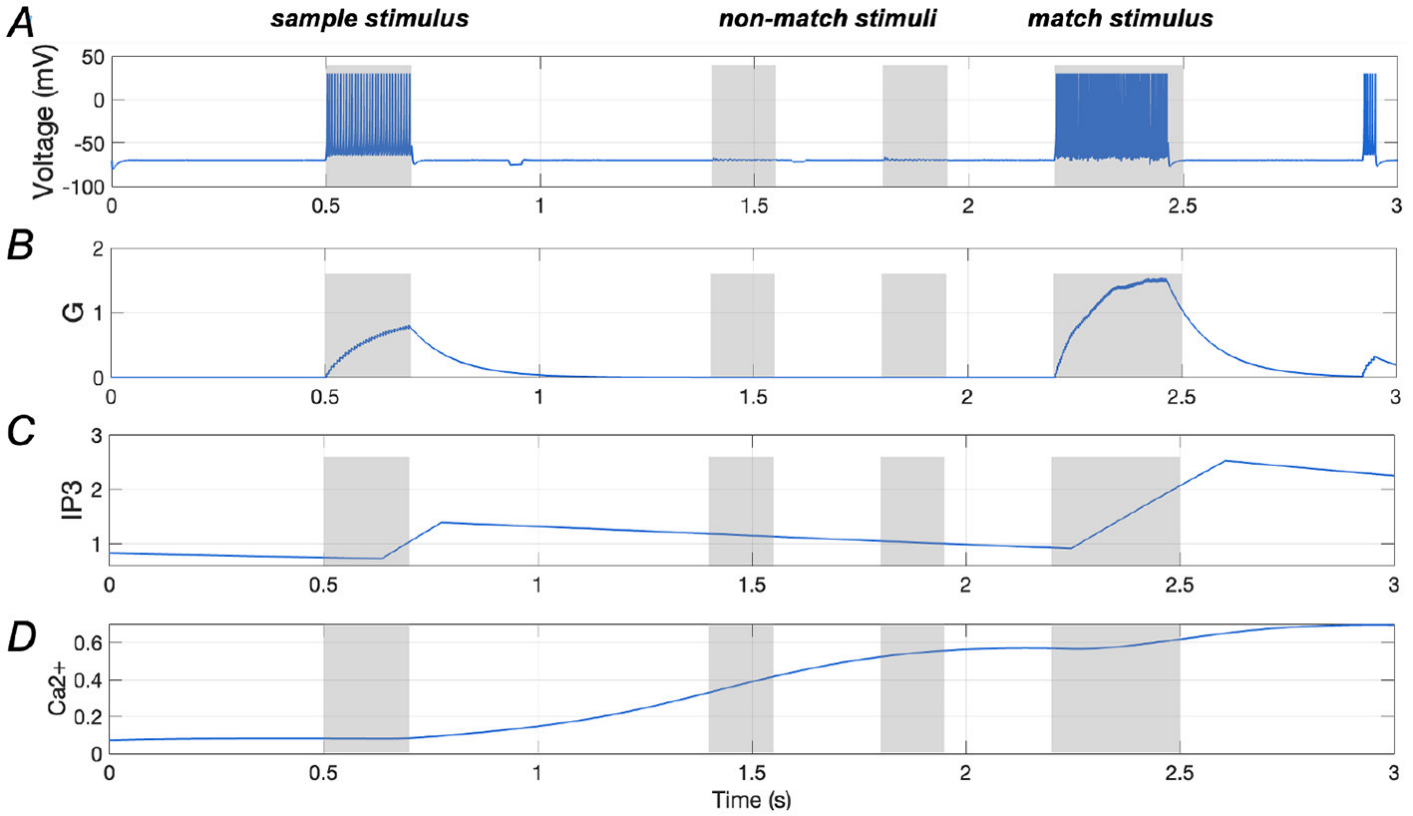
Responses of a stimulus-specific neuron and corresponding astrocyte. **(A, B)** Spike train of a specific neuron and the amount of glutamate (*G*) it releases, respectively; **(C, D)** Ca^2+^ signals and IP_3_ value of an astrocyte which has synaptic connection with the specific neuron.

Results presented in Figures 4A, B illustrate the spiking sequence of a stimulus-specific neuron along with the amount of glutamate (*G*) it releases, demonstrating comparable spiking activities for both the sample and match stimuli. Figures 4C, D display the *Ca*^2+^ signal and *IP*_3_ levels in an astrocyte that is that is synaptically connected to the stimulus-specific neuron. It is evident that once the glutamate release from the neuron reaches a certain threshold (“0.7”, as indicated in Equation 10), the *IP*_3_ level rises rapidly. This continuous increase in *IP*_3_ concentration gradually elevates the *Ca*^2+^ levels, ultimately enhancing neuronal responses. These findings highlight the critical role of *Ca*^2+^-mediated modulation in sustaining working memory, aligning with previous research (Mongillo et al., 2008).

### 3.2 WM network performance in the presence of **Aβ**

**Figure 5:**
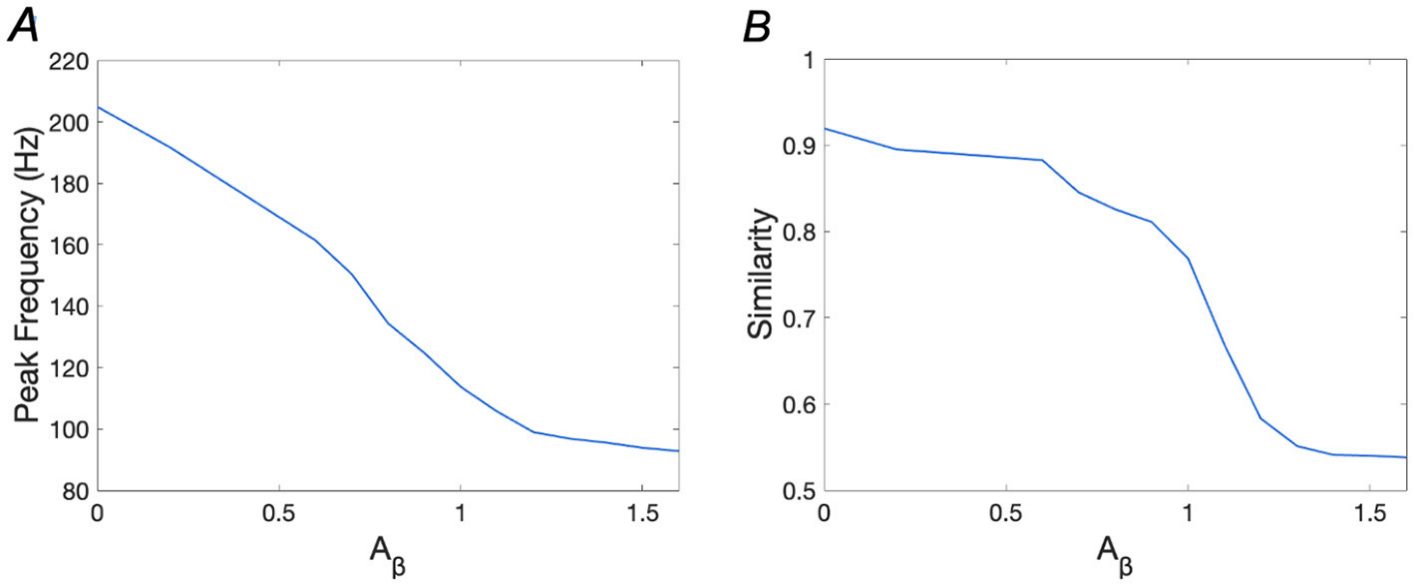
Peak frequencies and similarities with the increase of *A*β. **(A)** Peak Frequency vs. Aβ; **(B)** Similarity vs. Aβ.

Accumulation of *A*β has been linked to memory loss in AD in numerous studies (Hampel et al., 2021; Ma and Klann, 2012; Poling et al., 2008). In our research, we simulate *A*β accumulation by varying the *A*_β_ parameter and observe changes in memory performance. Figure 5 illustrates how similarity and peak frequency vary with respect to *A*β levels, showing that increased *A*β significantly impairs WM performance. Specifically, the peak frequency decreases from over 200 Hz (*A*_β_ = 0) to 93.87 Hz (*A*_β_ = 1.6), while similarity drops from 0.9195 (*A*_β_ = 0) to 0.541 (*A*_β_ = 1.6), indicating a substantial loss of memory recall fidelity.

In the following sections, we will separately analyze how memory performance is disrupted and the potential for restoration at *A*_β_ = 1.2 (mild AD) and *A*_β_ = 1.6 (severe AD).

#### 3.2.1 Working memory impairment under mild **Aβ** accumulation

**Figure 6:**
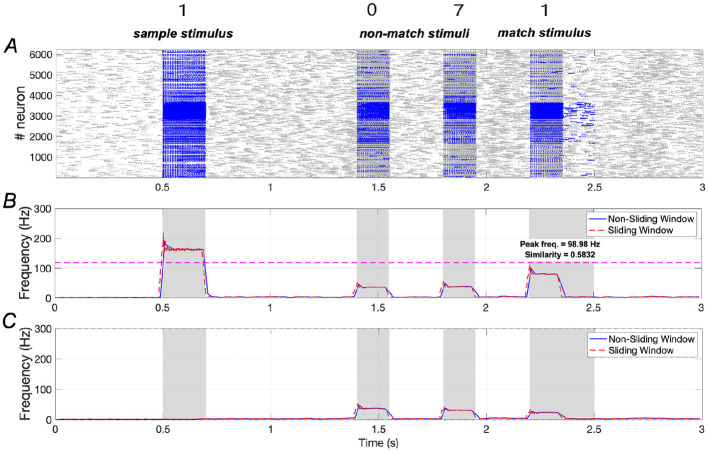
Neuronal responses in WM network in the presence of *A*β (*A*_β_ = 1.2). **(A)** Raster plots of spikes for all the neurons during different phases; **(B, C)** Average frequencies of stimulus-specific and unspecific neurons using different time windows, respectively (bin = 20 *ms*).

Results shown in Figure 6 indicate that the addition of a small amount of *A*β significantly impairs WM performance. Specifically, similarity decreases from 0.9195 to 0.5832, representing a reduction of ~36.6%, while peak frequency drops from 204.87 Hz to 98.98 Hz, reflecting a 51.7% reduction.

**Figure 7:**
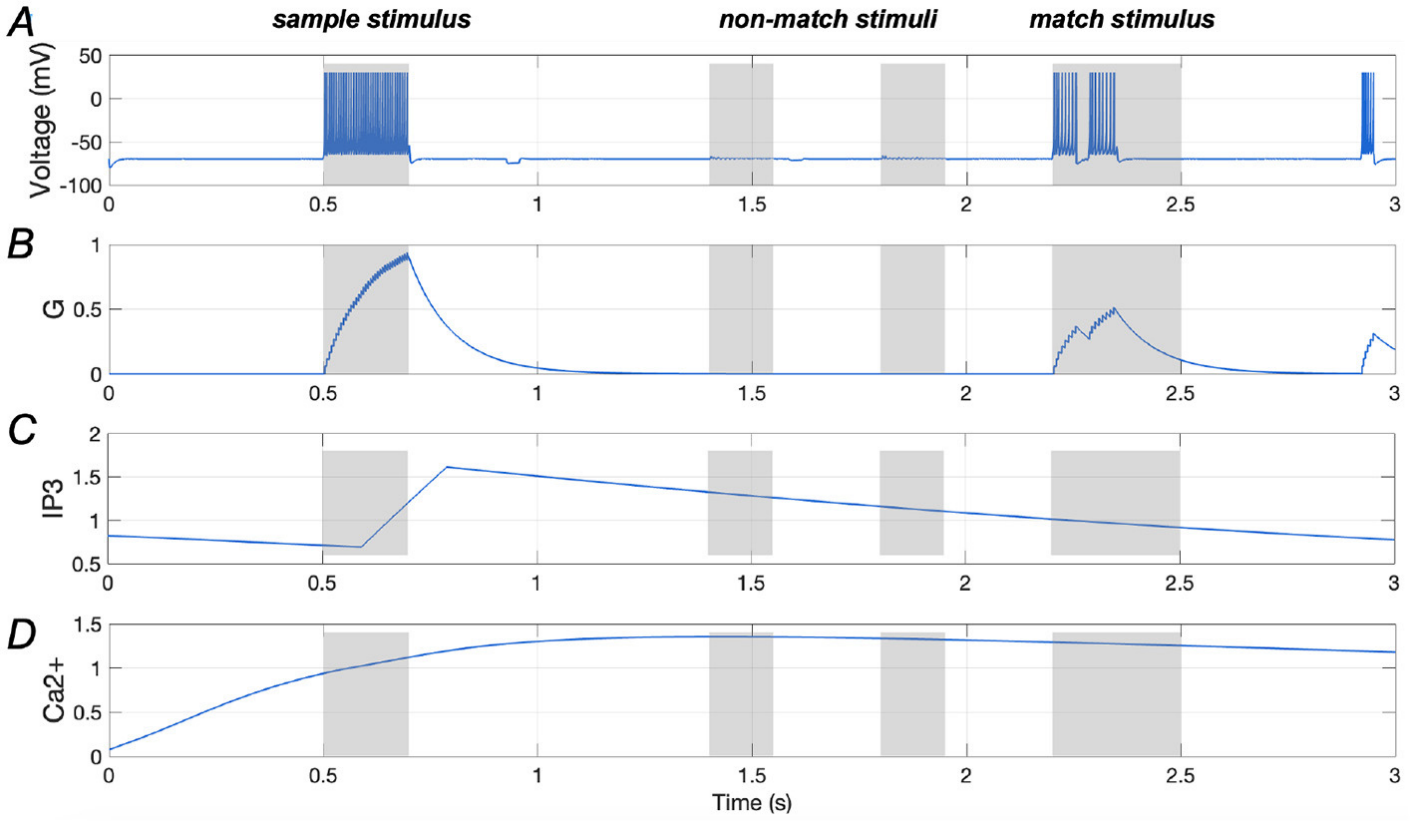
Responses of a stimulus-specific neuron and corresponding astrocyte in the presence of *A*β (*A*_β_ = 1.2). **(A, B)** Spike train of a specific neuron and the amount of glutamate (*G*) it releases, respectively; **(C, D)** *Ca*^2+^ signals and *IP*_3_ values of an astrocyte which has synaptic connection with the specific neuron.

Figures 7A, B illustrate the spiking response of a stimulus-specific neuron, and the amount of glutamate released, further highlighting the negative modulatory effect of *A*β. Additionally, the *Ca*^2+^ signal and IP3 levels in an astrocyte that is connected to the neuron are demonstrated in Figures 7C, D, in which the amplitude of *Ca*^2+^ signal shows an increasing trend, rising from about 0.7 (as seen in Figure 4D) to ~1.4 (as shown in Figure 7D). This finding aligns with previous studies indicating that *A*β accumulation disrupts calcium homeostasis, leading to increased *Ca*^2+^ concentrations in the intracellular space (Fani et al., 2021; Toglia et al., 2018).

#### 3.2.2 Working memory impairment under severe **Aβ** accumulation

**Figure 8:**
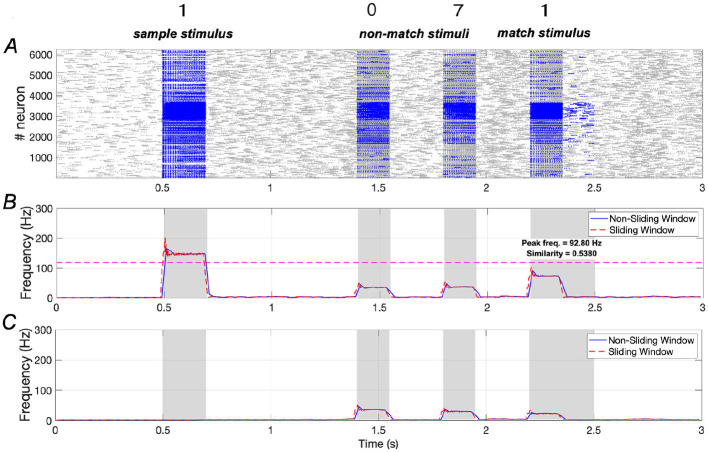
Neuronal responses in WM network in the presence of *A*β (*A*_β_ = 1.6). **(A)** Raster plots of spikes for all the neurons during different phases; **(B, C)** Average frequencies of stimulus-specific and unspecific neurons using different time windows, respectively (bin = 20 *ms*).

Results presented in Figure 8 indicate that the addition of a large amount of *A*β to the network severely impairs WM performance. Specifically, similarity decreases from 0.9195 to 0.5380, representing a reduction of ~41.5%, while peak frequency declines from 204.87 Hz to 92.80 Hz, reflecting a 54.7% reduction. These decreases are more pronounced than those observed at *A*_β_ = 1.2.

**Figure 9:**
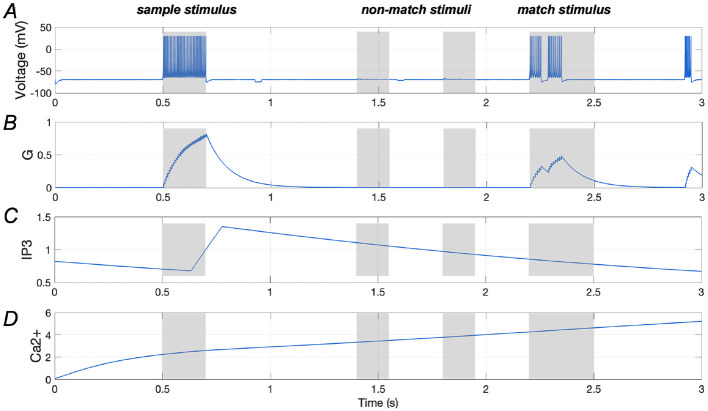
Responses of a stimulus-specific neuron and corresponding astrocyte in the presence of *A*β (*A*_β_ = 1.6). **(A, B)** Spike train of a specific neuron and the amount of glutamate (*G*) it releases, respectively; **(C, D)** Ca^2+^ signals and IP_3_ values of an astrocyte which has synaptic connection with the specific neuron.

Figures 9A, B illustrate the spiking response of a stimulus-specific neuron along with the amount of glutamate released, further emphasizing the negative modulatory effect of *A*β. Additionally, the *Ca*^2+^ signal and IP3 levels in an astrocyte that is connected to the neuron are demonstrated in Figures 9C, D, in which the amplitude of *Ca*^2+^ signal shows significant growth, reaching ~5 (as shown in Figure 9D).

### 3.3 Restoring WM network performance by downregulating **IP**_**3**_ activation

The results in Section 3.2 indicate that excessive levels of *Ca*^2+^ induced by *A*β play a crucial role in disrupting the memory performance of WM network. In this section, we investigate whether downregulation of *IP*_3_ can partially restore WM under pathological conditions by reducing intracellular calcium levels, as *IP*_3_ positively influences intracellular calcium concentration (see Equations 5, 6).

**Figure 10:**
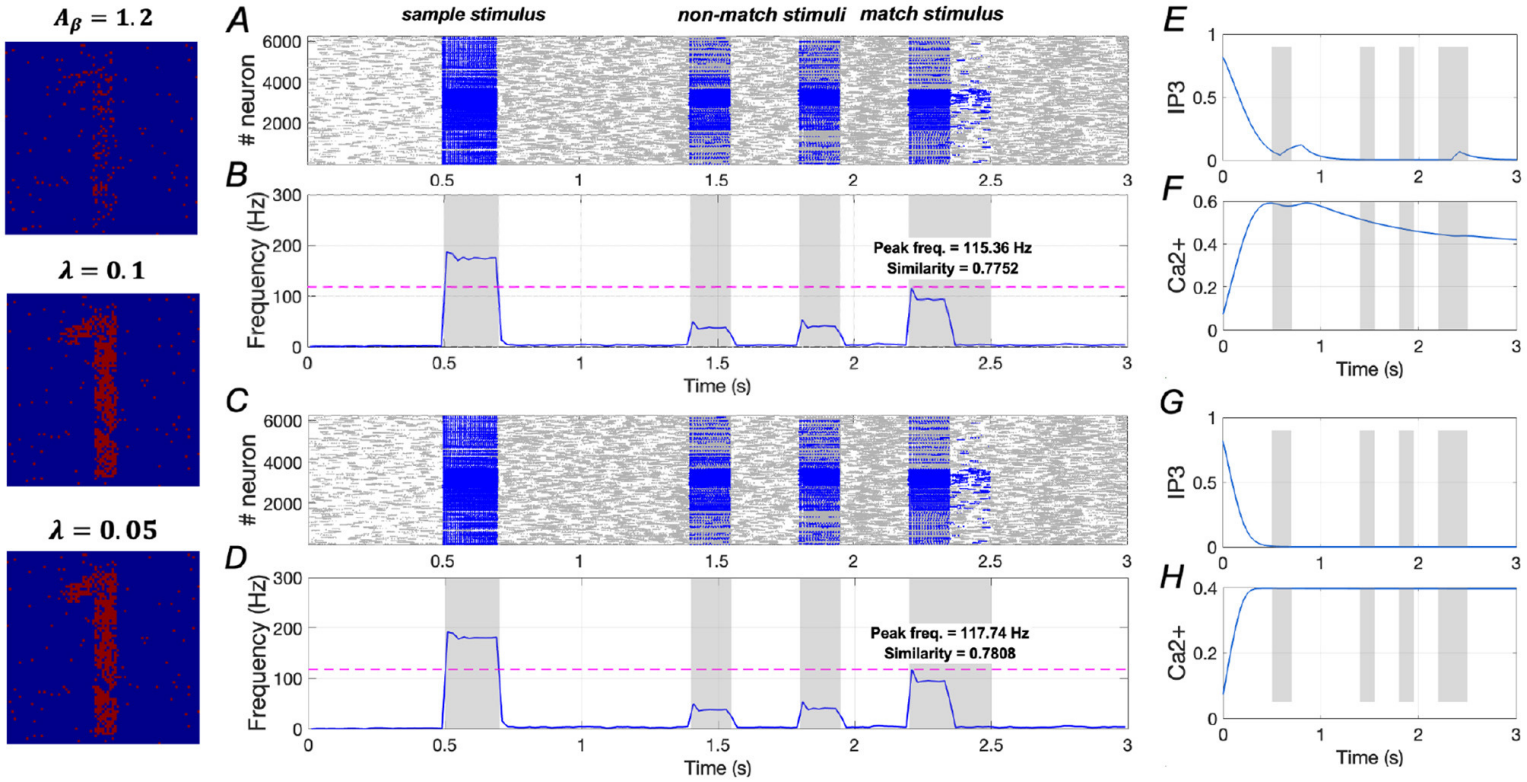
Modulation of memory performance by downregulating *IP*_3_ when *A*_β_ = 1.2. **(A, B)** and **(E, F)** Raster plot, average frequency of neurons, *Ca*^2+^ and *IP*_3_ of a specific astrocyte (λ = 0.1); **(C, D)** and **(G, H)** Raster plot, average frequency of neurons, *Ca*^2+^ and *IP*_3_ of a specific astrocyte (λ = 0.05).

In Figure 10, we analyze two cases under the condition of *A*_β_ = 1.2 (mild AD): (1) When λ = 0.1 (see Equation 7), similarity increases from 0.5832 to 0.7752, a rise of ~32.9%, and peak frequency increases from 98.98 Hz to 115.36 Hz, reflecting a 16.5% increase; (2) When λ = 0.05, similarity rises from 0.5832 to 0.7808, representing a 33.9% increase, while peak frequency increases from 98.98 Hz to 117.74 Hz, showing a 19.0% increase. These findings suggest that downregulation of *IP*_3_ activation can significantly enhance WM performance under mild AD conditions.

**Figure 11:**
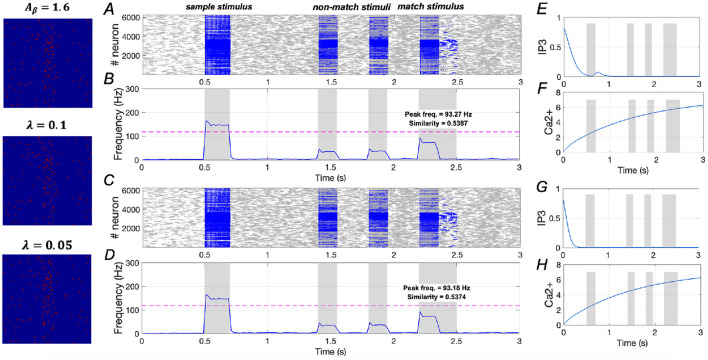
Modulation of memory performance by downregulating *IP*_3_ when *A*_β_ = 1.6. **(A, B)** and **(E, F)** Raster plot, average frequency of neurons, *Ca*^2+^ and *IP*_3_ of a specific astrocyte (λ = 0.1); **(C, D)** and **(G, H)** Raster plot, average frequency of neurons, *Ca*^2+^ and *IP*_3_ of a specific astrocyte (λ = 0.05).

However, in Figure 11, under the condition of *A*_β_ = 1.6 (severe AD), both λ = 0.1 and λ = 0.05 show minimal effects on similarity and peak frequency. This indicates that downregulating *IP*_3_ activation does not improve WM performance in severe AD, underscoring the necessity for a dual-target modulation strategy to achieve full recovery in severe AD patients.

### 3.4 Restoring WM network performance by downregulating **IP**_**3**_ activation and upregulating **SERCA** activation

**Figure 12:**
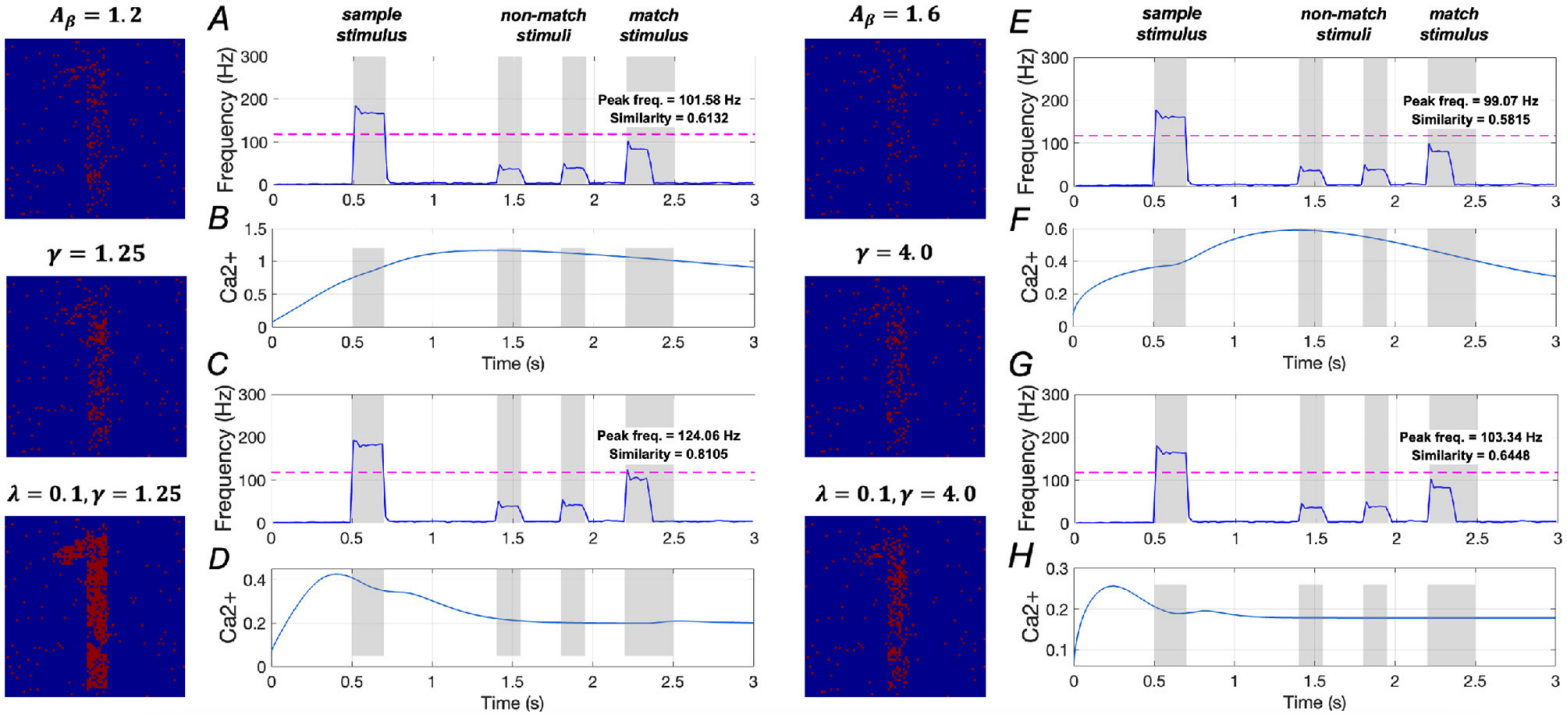
Modulation of memory performance by downregulating *IP*_3_ and upregulating *SERCA* activations. Average frequency of neurons and *Ca*^2+^ signal of a specific astrocyte: **(A, B)** γ = 1.25; **(C, D)** λ = 0.1, γ = 1.25; **(E, F)** γ = 4.0; **(G, H)** λ = 0.1, γ = 4.0.

The effects of upregulating *SERCA* activation on WM performance are illustrated in Figure 12. In mild AD conditions, when γ = 1.25, similarity increases from 0.5832 to 0.6132, reflecting a ~5.1% increase, and peak frequency rises from 98.98 Hz to 101.58 Hz, a ~2.6% increase. However, when γ = 2.0, similarity increases from 0.5832 to 0.7710, representing a ~32.2% increase, while peak frequency rises from 98.98 Hz to 114.06 Hz, a ~ 15.2% increase (see Supplementary Figure 19). The corresponding variations in the *Ca*^2+^ signal for γ = 1.25 is shown in Figure 12B.

Moreover, simultaneously altering the activations of *IP*_3_ and *SERCA* significantly enhances WM performance. Specifically, similarity rises from 0.5832 to 0.8105, a ~39.0% increase, and peak frequency increases from 98.98 Hz to 124.06 Hz, reflecting a ~25.3% increase. These findings suggest that concurrently downregulating *IP*_3_ activation while upregulating *SERCA* activation plays a crucial role in restoring WM under mild AD conditions.

In Figures 12E–H, the model results for severe AD conditions indicate that upregulating *SERCA* activation has minimal impact on WM performance. Additionally, simultaneous changes to the activations of *IP*_3_ and *SERCA* show only slight improvements in WM performance. These results imply that simply regulating intracellular calcium levels is insufficient to effectively restore WM performance in severe AD conditions.

### 3.5 Test the modulatory roles of **IP**_**3**_ and **SERCA** using other two sample stimuli

To evaluate the general applicability of our network model, we conducted two additional experiments by changing the sample stimulus to two other digit stimuli: “2” and “7”.

#### 3.5.1 Model evaluation using digit “2” as sample stimulus

**Figure 13:**
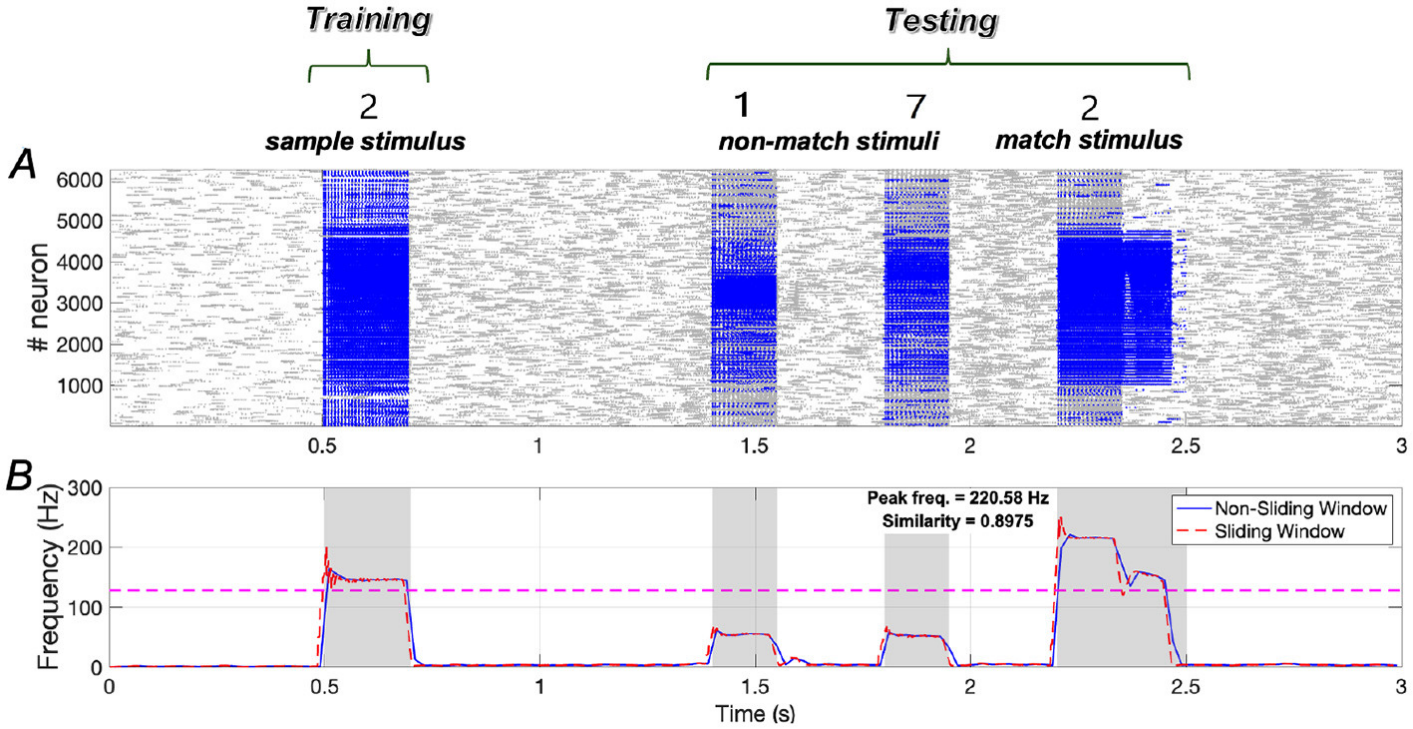
Neuronal responses in the WM network when the sample stimulus image is “2”. **(A)** Raster plots of spikes for all the neurons during different phases; **(B)** Average frequencies of stimulus-specific neurons using different time windows, respectively (bin = 20 *ms*).

Results demonstrated in Figure 13 indicate that the similarity of neuronal responses to the sample and match stimuli reaches ~0.8975, with the peak frequency of neurons responding to the match stimulus being comparable to that of neurons responding to the sample stimulus.

**Figure 14:**
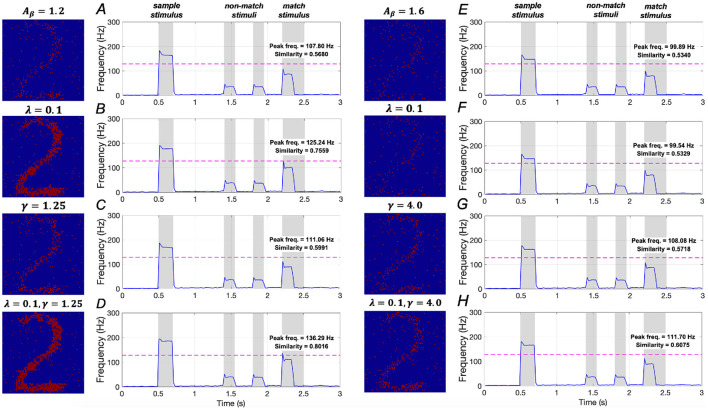
Modulation of memory performance when the sample stimulus image is “2”. Average frequency of neurons: **(A)** AD condition (*A*_β_ = 1.2); **(B)** λ = 0.1; **(C)** γ = 1.25; **(D)** λ = 0.1, γ = 1.25; **(E)** AD condition (*A*_β_ = 1.6); **(F)** λ = 0.1; **(G)** γ = 4.0; **(H)** λ = 0.1, γ = 4.0.

The effects of downregulating *IP*_3_ activation and upregulating *SERCA* activation on WM performance are illustrated in Figures 14A–D. In mild AD conditions, when λ = 0.1, similarity increases from 0.5680 to 0.7559, representing a ~33.1% increase, and peak frequency rises from 107.80 Hz to 125.24 Hz, a ~16.2% increase. When γ = 1.25, similarity increases from 0.5680 to 0.5991 (~5.5 increase), and peak frequency rises from 107.80 Hz to 111.06 Hz (~3.0% increase). When both λ = 0.1 and γ = 1.25 are applied, similarity increases from 0.5680 to 0.8016 (~41.1%), and peak frequency rises from 107.80 Hz to 136.29 Hz (~26.4%). These findings suggest that simultaneously downregulating *IP*_3_ activation and upregulating *SERCA* activation plays a crucial role in restoring WM under mild AD conditions, consistent with the results observed under the sample stimulus “1”.

In severe AD conditions, the model results shown in Figures 14E–H demonstrate that manipulating the activation of *IP*_3_ and *SERCA*, either separately or simultaneously, does not significantly improve memory performance. These findings imply that the WM performance in severe AD cannot be effectively restored solely by regulating intracellular calcium levels, aligning with the conclusions drawn under the sample stimulus “1”.

#### 3.5.2 Model evaluation using digit “7” as sample stimulus

**Figure 15:**
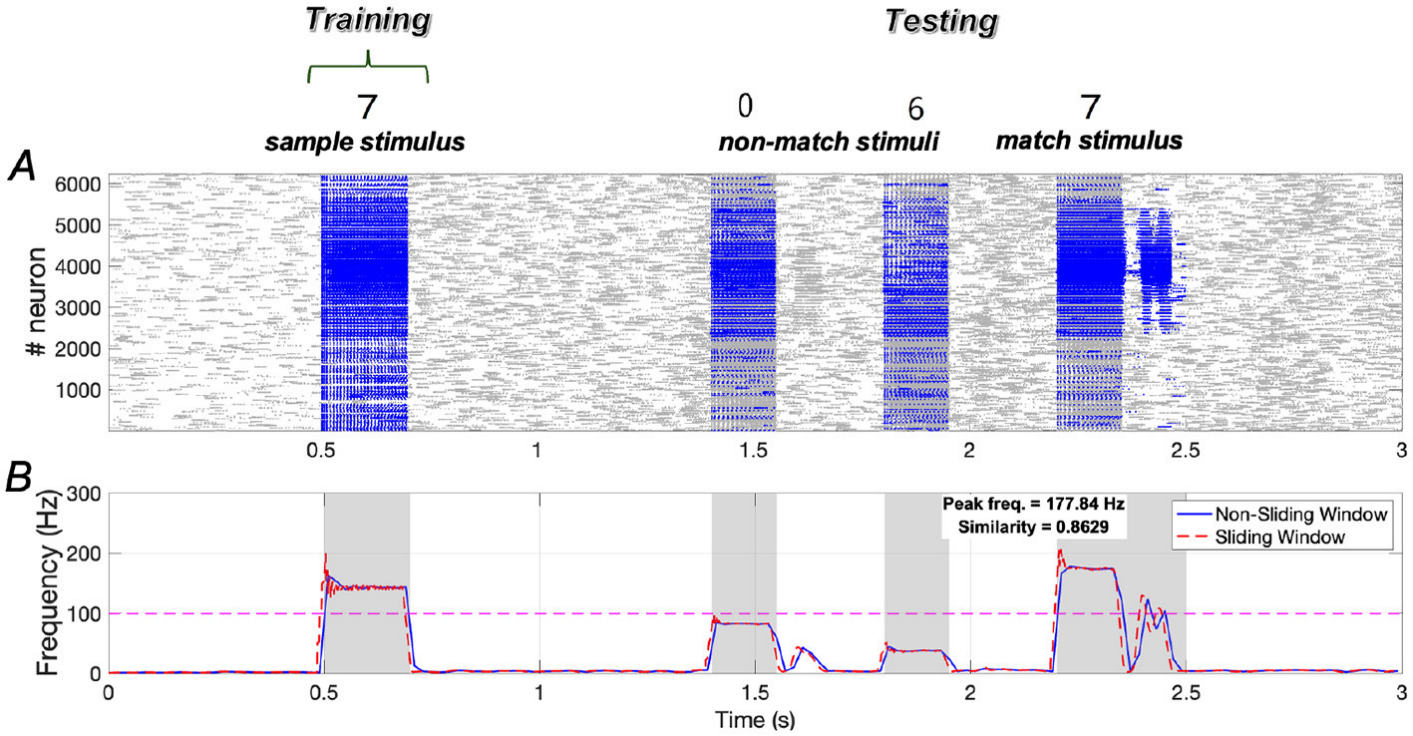
Neuronal responses in the WM network when the sample stimulus image “7”. **(A)** Raster plots of spikes for all the neurons during different phases; **(B)** Average frequencies of stimulus-specific neurons using different time windows, respectively (bin = 20 *ms*).

The trends in similarity and peak frequency under sample stimulus “7” are consistent with those observed for sample stimuli “1” and “2”. Specifically, under sample stimulus “7”, the similarity of neuronal responses to the sample and match stimuli reaches ~0.8629, with the peak frequency of neurons responding to the match stimulus being comparable to that of neurons responding to the sample stimulus (Figure 15).

**Figure 16:**
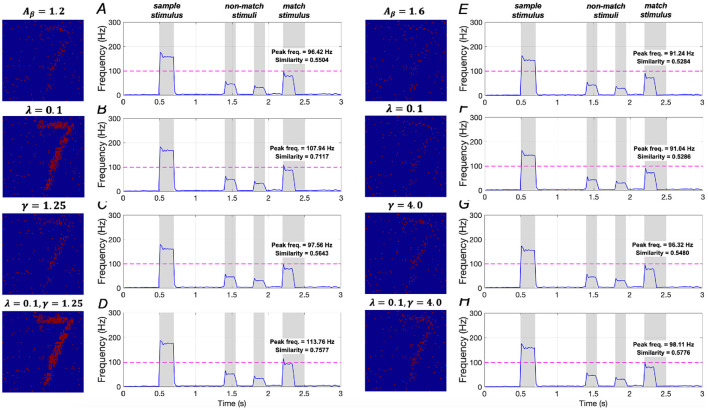
Modulation of memory performance when the sample stimulus image is “7”. Average frequency of neurons: **(A)** AD condition (*A*_β_ = 1.2); **(B)** λ = 0.1; **(C)** γ = 1.25; **(D)** λ = 0.1, γ = 1.25; **(E)** AD condition (*A*_β_ = 1.6); **(F)** λ = 0.1; **(G)** γ = 4.0; **(H)** λ = 0.1, γ = 4.0.

The effects of downregulating *IP*_3_ activation and upregulating *SERCA* activation on WM performance are illustrated in Figures 16A–D. In mild AD conditions, when λ = 0.1, similarity increases by ~29.3% (from 0.5504 to 0.7117), and peak frequency shows an ~11.9% increase (from 96.42 Hz to 107.94 Hz). When γ = 1.25, there is a small increase of ~2.5% in similarity (from 0.5504 to 0.5643) and ~1.2% in peak frequency (from 96.42 Hz to 97.56 Hz). When both λ = 0.1 and γ = 1.25 are applied, similarity increases significantly by ~37.7% (from 0.5504 to 0.7577), and peak frequency rises by ~18.0% (from 96.42 Hz to 113.76 Hz). These results suggest that simultaneously downregulating *IP*_3_ activation and upregulating *SERCA* activation plays a crucial role in restoring WM in mild AD conditions, consistent with the findings under sample stimulus “1”.

Model results for severe AD conditions, shown in Figures 16E–H, demonstrate that manipulating the activations of *IP*_3_ and *SERCA*, either separately or together, does not significantly impact memory performance. There results indicating that WM performance in severe AD cannot be effectively restored by merely regulating intracellular calcium levels, aligning with the conclusions drawn from sample stimulus “1”.

## 4 Discussions

In this study, we developed a computational model comprising spiking neurons and non-spiking astrocytes to explore how intracellular calcium homeostasis affects WM performance under *A*β-induced AD conditions. Quantitative measures, represented by similarity and peak frequency, indicate that manipulating *IP*_3_ receptor activation and *SERCA* pump activity can significantly enhance memory performance in mild AD conditions. However, minimal effects on performance are observed in severe AD conditions when altering *IP*_3_ and *SERCA* activation. Evaluations using two additional sample stimuli further confirm the general applicability of our network model.

Several previous experimental findings support our proposed modulation strategies of downregulating *IP*_3_ and upregulating *SERCA* as physiologically realistic (Green et al., 2008; Krajnak and Dahl, 2018; Kumar et al., 2015). For instance, mGluR5 antagonists, such as MPEP and fenobam, can pharmacologically reduce *IP*_3_ production by blocking metabotropic glutamate receptor 5 (Kumar et al., 2015). These drugs have been shown to effectively reduce synaptic hyperexcitability and improve memory deficits in mouse models of AD, aligning with our model predictions. Additionally, enhancing *SERCA* pump efficiency has been explored through *SERCA*2*a* gene therapy in cardiac disease models (Kranias and Hajjar, 2012), where improved calcium handling restores cellular function. While *SERCA* upregulation has not been extensively tested in neuronal contexts, pharmacological activators and gene delivery vectors present promising avenues. Together, these strategies offer a translationally relevant approach to restore calcium homeostasis and potentially reverse early WM damage in *A*β-induced mild AD conditions.

Despite the effectiveness of our model in characterizing WM formation and restoration, several limitations still need to be addressed.

### 4.1 Model components in the network

First, our network model includes only excitatory neurons, overlooking the crucial role of inhibitory interneurons, which are essential for synchronizing network rhythms and preventing overexcitability. Future studies should incorporate inhibitory interneurons to enhance the realism of the memory model. Second, the astrocyte model is based on mean-field approximations, averaging calcium responses across compartments (Bazargani and Attwell, 2016). This approach simplifies the intricate spatial signaling seen in actual astrocytes, particularly in presynaptic domains. Integrating spatially distinct, cell-type-specific mechanisms would enhance the model's fidelity and biological relevance for future laboratory research.

### 4.2 **Aβ** and other hypotheses

In recent decades, the pathogenesis of AD has received considerable attention, leading to several prominent hypotheses: (1) Amyloid Hypothesis (*A*β): This hypothesis suggests that neuronal damage in AD patients arises from abnormalities in the processing of amyloid precursor protein (APP) and the subsequent accumulation of *A*β (Hampel et al., 2021). (2) Cholinergic Hypothesis: Supported by experimental observations, this hypothesis highlights a significant reduction in choline acetyltransferase, the enzyme responsible for synthesizing acetylcholine (ACh), in the amygdala, cortex, and hippocampus of postmortem brains from AD patients (Chen et al., 2022). (3) Glutamate Toxicity Hypothesis: This hypothesis is based on evidence of a marked reduction in the binding of 1-[3H] glutamate in the brains of individuals with AD (Maragos et al., 1987). (4) Tau Hypothesis: This hypothesis is grounded in the observation of aggregates of misfolded tau proteins present in the brains of AD patients (Frost et al., 2009).

While these four hypotheses aim to elucidate the pathophysiological basis of AD, it is important to recognize that the triggers associated with these hypotheses likely do not operate in isolation and may interact synergistically (Patow et al., 2023). In this study, we focused solely on AD conditions induced by *A*β accumulation. Future research should also examine AD conditions triggered by other significant factors.

### 4.3 Synaptic plasticity in WM network

Plasticity is a well-documented phenomenon at nearly all synapses in the brain, encompassing various types such as short-term plasticity, long-term plasticity (including long-term potentiation and long-term depression), Hebbian learning, and spike-timing-dependent plasticity (STDP). Moreover, synaptic plasticity is believed to play a critical role in the formation and storage of WM (Froudist-Walsh et al., 2018; Huang and Wei, 2021). In this study, however, the performance of the WM network is driven solely by intracellular calcium dynamics, without incorporating mechanisms of synaptic plasticity. Therefore, a potential extension of our network model could involve integrating plasticity mechanisms to explore changes in synaptic weights among cells.

### 4.4 Functions of **Aβ** at the genetic level

Our network model operates at the cellular level, while the specific mechanisms of *A*_β_ function predominantly occur at the genetic level. For example, Hao and Friedman developed a mathematical model that outlines the *A*β aggregation process in detail, including production, clearance, and degradation (Hao and Friedman, 2016). Mustafa et al. created a two-compartment diffusion model to examine the effects of *A*β on the acetylcholine neurocycle, incorporating a differential equation to characterize changes in *A*β levels (Mustafa et al., 2021). Similarly, Chamberland et al. proposed a multiscale model consisting of 19 ordinary differential equations to analyze the progression of AD, introducing equations for both intracellular and extracellular *A*β (Chamberland et al., 2024). Models that focus on *A*β at the genetic level may offer a more accurate representation of its dynamic variations. Therefore, future modeling studies on *A*β-related AD generation and progression should incorporate more genetic-level components.

### 4.5 Other types of memory impairments

Memory impairments are common symptoms observed in the brains of AD patients. Alongside deficits in WM, other types of memory, such as short-term, episodic, and semantic memories (Jahn, 2013), are also affected during the progression of AD. Furthermore, various memory-related symptoms, including sleep disturbances (Pathmanathan et al., 2025) and accelerated long-term forgetting (Stamate et al., 2020), have been observed. Recent years have seen the development of computational models addressing these symptoms. For example, Horn et al. constructed a biologically motivated Hopfield model to investigate how the interplay between synaptic deletion and compensation influences memory deterioration patterns (Horn et al., 1993). Razi et al. built a network model of coupled cortical columns to explore the propagation modes between sleep and wakefulness (Razi et al., 2021). Additionally, Murre et al. introduced a mathematical model to analyze the dynamics of forgetting and amnesia, applying their findings to experimental observations from mice, rats and monkeys (Murre et al., 2013). Given this context, future computational studies could integrate these model explorations with our spiking network model to investigate potential therapeutic targets for these symptoms under AD conditions.

## Data Availability

The original contributions presented in the study are included in the article/Supplementary material, further inquiries can be directed to the corresponding author.
