# Supplementary material for: The role of IP3 receptors and SERCA pumps in restoring working memory under amyloid β induced Alzheimer's disease: a modeling study
Source: Front Comput Neurosci. 2025 Jul 22;19:1643547. doi: 10.3389/fncom.2025.1643547 (PMC12321881; doi:10.3389/fncom.2025.1643547)
Supplement: Supplementary file 1 [file Data_Sheet_1.pdf]

Supplementary Figures

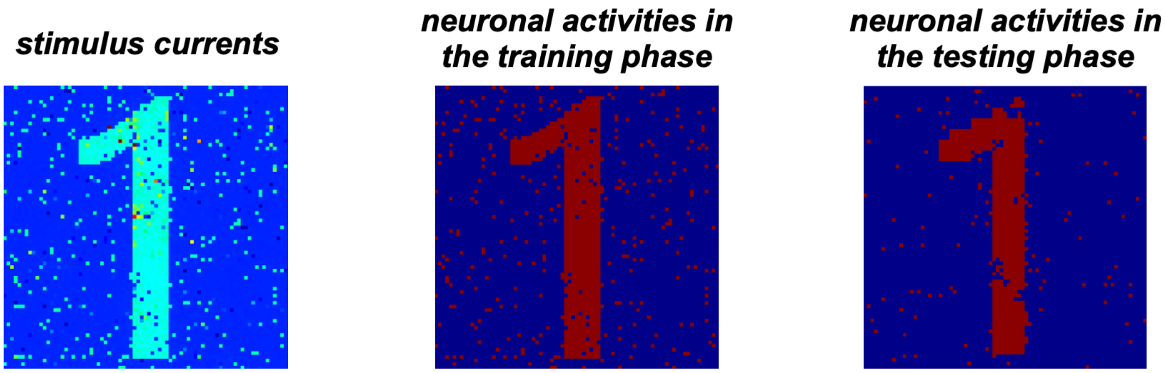

Figure 17 Demonstration of the stimulus currents, neuronal activities in the training and testing phases.

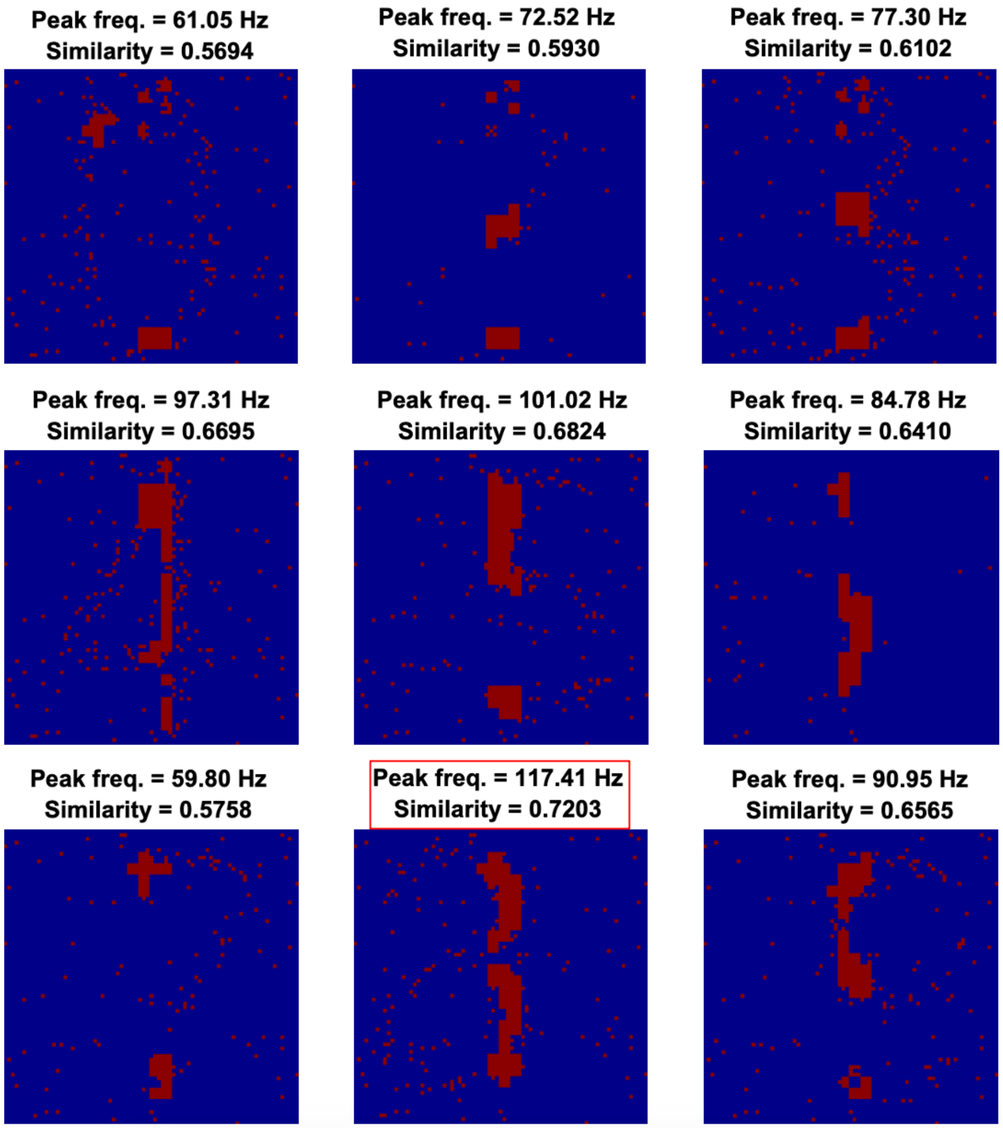

Figure 18 Peak frequencies and similarities when the match stimulus during the testing phase is: "0", "2", "3", "4", "5", "6", "7", "8", and "9".

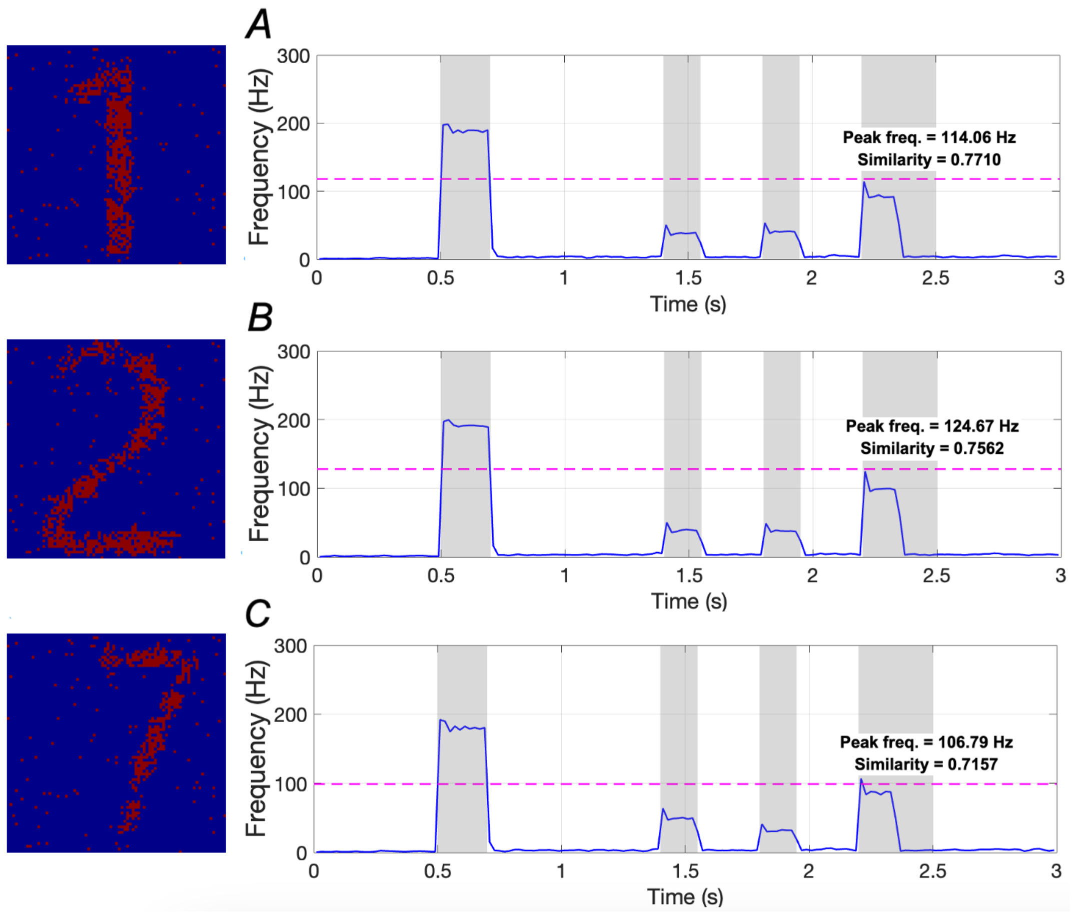

Figure 19 Modulation of memory performance by upregulating $SERCA$ ($\gamma = 2.0$). Average frequency of neurons: (A) under sample stimulus "1"; (B) under sample stimulus "2"; (C) under sample stimulus "7".
